# Tumor necroptosis-mediated shedding of cell surface proteins promotes metastasis of breast cancer by suppressing anti-tumor immunity

**DOI:** 10.1186/s13058-023-01604-9

**Published:** 2023-01-26

**Authors:** Zhaoshan Liu, Swati Choksi, Hyung-Joon Kwon, Delong Jiao, Chengyu Liu, Zheng-gang Liu

**Affiliations:** 1 Laboratory of Immune Cell Biology, Center for Cancer Research, National Cancer Institute, National Institutes of Health, Bethesda, MD 20892 USA; 2 Transgenic Core, National Heart Lung and Blood Institute, National Institutes of Health, Bethesda, MD 20892 USA

**Keywords:** Necroptosis, Breast cancer, Metastasis, MLKL, ADAMs

## Abstract

Necroptosis is a form of regulated necrosis and is executed by MLKL when MLKL is engaged in triggering the rupture of cell plasma membrane. MLKL activation also leads to the protease, ADAMs-mediated ectodomain shedding of cell surface proteins of necroptotic cells. Tumor necroptosis often happens in advanced solid tumors, and blocking necroptosis by MLKL deletion in breast cancer dramatically reduces tumor metastasis. It has been suggested that tumor necroptosis affects tumor progression through modulating the tumor microenvironment. However, the exact mechanism by which tumor necroptosis promotes tumor metastasis remains elusive. Here, we report that the ectodomain shedding of cell surface proteins of necroptotic cells is critical for the promoting effect of tumor necroptosis in tumor metastasis through inhibiting the anti-tumor activity of T cells. We found that blocking tumor necroptosis by MLKL deletion led to the dramatic reduction of tumor metastasis and significantly elevated anti-tumor activity of tumor-infiltrating and peripheral blood T cells. Importantly, the increased anti-tumor activity of T cells is a key cause for the reduced metastasis as the depletion of CD8+ T cells completely restored the level of metastasis in the Mlkl KO mice. Interestingly, the levels of some soluble cell surface proteins including sE-cadherin that are known to promote metastasis are also dramatically reduced in MLKL null tumors/mice. Administration of ADAMs pan inhibitor reduces the levels of soluble cell surface proteins in WT tumors/mice and leads to the dramatic decrease in metastasis. Finally, we showed the sE-cadherin/KLRG1 inhibitory receptor is the major pathway for necroptosis-mediated suppression of the anti-tumor activity of T cells and the promotion of metastasis. Hence, our study reveals a novel mechanism of tumor necroptosis-mediated promotion of metastasis and suggests that tumor necroptosis and necroptosis-activated ADAMs are potential targets for controlling metastasis.

**Supplementary Information:**

The online version contains supplementary material available at 10.1186/s13058-023-01604-9.

## Background

Necroptosis is a form of programmed necrotic cell death [1–3]. While it is first reported through studying death receptor-induced cell death [4], necroptosis mostly happens under pathological conditions including viral infection in vivo. For death-receptor-induced necroptosis, the protein kinase receptor-interacting protein kinase 1, 3 (RIPK1, RIPK3) and mixed lineage kinase domain-like protein (MLKL) are the key components of the necroptosis machinery [1–3]. When the activity of cellular inhibitor of apoptosis proteins (cIAPs) and Caspase-8 are inhibited in cells, the engagement of death receptor triggers RIPK1 to recruit RIPK3, which in turn recruits MLKL to form the death complex known as necrosome, to initiate necroptosis [5–10]. In the necrosome, RIPK3 is autophosphorylated and subsequently, the activated RIPK3 recruits and phosphorylates MLKL [9–11]. Then, MLKL oligomerizes and translocates to the plasma membrane to carry out the execution of necroptosis [12–15]. For viral infection-induced necroptosis, RIPK3 and MLKL, but not RIPK1, are required and another protein, Z-DNA-binding protein 1 (ZBP1), also known as DNA-dependent activator of IFN regulatory factors (DAI), functions upstream of RIPK3-MLKL to initiate the formation of the necrosome [16]. As a necrotic cell death, the rupture of cell plasma membrane of necroptotic cells results in the release of many cellular factors of necroptotic cells, which may trigger inflammation and immune responses [17, 18]. Importantly, our recent study demonstrated that ZBP1, not RIPK1, mediates tumor necroptosis in breast cancer [19].

The translocation of the oligomerized, phosphorylated MLKL also leads to the activation of the cell surface proteases, a disintegrin and metalloproteases (ADAMs), which cause ectodomain shedding of many cell surface proteins of necroptotic cells including cadherins, ErbB2, epithelial cellular adhesion molecule (EpCAM) and many others [20, 21]. The shedding of cell surface proteins by ADAMs promotes necroptosis, cell migration and inflammation [21]. Notably, the soluble forms of some of these proteins, such as E-cadherin, junctional adhesion molecule A (JAM-A) and syndecan-1, have been shown to play a key role in tumor progression by modulating tumor microenvironments and high levels of these proteins in the serum are an indicator of poor prognosis [22–26]. For example, the circulating level of soluble E-cadherin (sE-cad) is known to be highly increased in cancer patients and is correlated with tumor metastasis [26, 27]. Also, the soluble E-cadherin is a known ligand of the inhibitory receptor, killer cell lectin-like receptor subfamily G member 1 (KLRG1) on lymphocytes, and this interaction has been suggested to inhibit T cell activity [28, 29].

In our previous studies, we found that tumor necroptosis promotes lung metastasis [30], but, however, the underlying mechanism for the promoting effect of necroptosis on tumorigenesis is largely unknown. As it is well documented that the inhibition of the tumor-suppressing functions of T cells plays a key role in tumor metastasis [31, 32], we investigated whether tumor necroptosis inhibits the anti-tumor functions of T cells in two preclinical mouse breast cancer models, a genetic modified MMTV-PyMT model and a orthotopic transplantation MVT-1 model [33, 34]. In this study, we demonstrated that the ectodomain shedding of cell surface proteins of necroptotic cells promotes tumor metastasis through inhibiting the anti-tumor activity of T cells. We found that blocking tumor necroptosis by MLKL deletion in both MMTV-PyMT mice and MVT-1 tumors resulted in the dramatic reduction of tumor metastasis to the lungs and the increase in the anti-tumor activity of both tumor-infiltrating and peripheral blood T cells. We found that the levels of soluble cell surface proteins, E-cadherin, JAM-A, syndecan-1, are dramatically reduced in MLKL null tumors/mice. Importantly, we showed that the administration of the ADAMs pan inhibitor in mice with WT tumors reduces the levels of all these three soluble proteins and leads to the increased anti-tumor activity of T cells and the dramatic decrease in metastasis. Finally, we showed that sE-cad/KLRG1 pathway plays a major role in mediating necroptosis-triggered inhibition of the anti-tumor activity of T cells. Hence, our study reveals a novel mechanism of tumor necroptosis-promoted metastasis and suggests that tumor necroptosis is a potential target for controlling metastasis.

## Methods

### Reagents and antibodies

Anti-phospho-MLKL (ab196436), anti-MLKL (ab184718), anti-GSDMD (ab209845) antibodies from Abcam; anti-cleaved Caspase3 (9664), anti-Caspase3 (9662), anti-cleaved Caspase8 (8592), anti-Caspase8 (4927), anti-cleaved Caspase9 (9509), anti-Caspase9 (9508) antibodies from Cell signaling; β-actin (A3853) from Sigma. Anti-KLRG1 antibody (16–5893-85) and IgG isotype control (16–4914-85) from eBioscience. Anti-CD8 antibody (BP0117) and IgG isotype control (BP0090) from Bioxcell. GW280264X (AOB3632) from AOBIOUS.

### Mice

FVB/NJ and MMTV-PyMT mice were purchased from The Jackson Laboratory. All animal experiments were performed under protocols approved by the National Cancer Institute Animal Care and Use Committee and followed NIH guidelines. For orthotopic model, MVT-1 cells (syngeneic mouse mammary cancer cell line derived from c-Myc/VEGF tumor explants) were suspended in 100 µl Matrigel Matrix (Corning) solution (diluted 1:1 with PBS) and then injected (2 × 10^6^/mouse) into the right inguinal mammary fat pad of FVB/NJ mice. Tumor volume was monitored weekly. For Anti-KLRG1 antibody injection, the mice were injected intraperitoneally with Anti-KLRG1 antibody or IgG isotype at 12 weeks until 15 weeks by every 3 days (1 mg/kg). For Anti-CD8 antibody injection, the mice were injected intraperitoneally with Anti-CD8 antibody or IgG isotype at 12 weeks until 15 weeks every 5 days (500ug/mouse/injection). For GW280264X injection, the mice were injected intraperitoneally with vehicle (3% DMSO in PBS) or GW280264X (3ug/mouse/injection) at 12 weeks until 15 weeks every 3 days. To be comparable to the late stage necrotic MMTV-PyMT and MVT-1 tumors, the largest tumor from these models was collected as indicated for further analysis.

### Generation of Mlkl knockout and MMTV-PyMT double transgenic mice

The Mlkl knockout mice were generated using the CRISPR/Cas9 method. Briefly, two single guide RNAs (sgRNAs) were designed to target the first coding exon of the mouse Mlkl gene, one (TTGGGACAGATCATCAAGTT) targeting shortly after the translation initiation codon (ATG) and the other one (GCACACGGTTTCCTAGACGC, in reverse orientation) targeting after the only other Met (in frame ATG) in this exon for eliminating its possibility of being used as an alternative translation initiation codon to make a truncated protein. The two sgRNA DNA constructs were made using OriGene’s gRNA Cloning Services (Rockville, Maryland) and were then used as templates to synthesize sgRNAs using MEGAshortscript T7 Kit (Invitrogen). The two sgRNAs (20ug/ml each) were co-microinjected with Cas9 mRNA (100ug/ml, purchased from TriLink BioTechnologies) into the cytoplasm of fertilized mouse eggs, which were collected from mating pairs between male MMTV-PyMT transgenic mice (JAX Stock # 002374) and female FVB/NJ mice (JAX Stock #001800). The injected zygotes were cultured overnight in M16 medium (Millipore Sigma) at 37 °C in 6% CO_2_. Those embryos reached 2-cell stage of development were implanted into the oviducts of pseudopregnant surrogate mothers. Mice born to these foster mothers were genotyped by PCR amplification of the region surrounding the CRISPR cutting sites followed by Sanger DNA sequencing. Founder mice with frameshift deletions were bred with MMTV-PyMT mice to establish Mlkl KO and MMTV-PyMT double transgenic lines. For use in experiments, MMTV-PyMT/MLKL heterozygous male mice were bred with female MLKL heterozygous mice to obtain littermates MLKL wild type and Mlkl KO carrying MMTV-PyMT. To verify Mlkl genotype by PCR, the following primers were used: Forward 5′-TATGGATAAATTGGGACA-3′, Reverse: 5′-CGTCTTCCTCTGCATCCT-3′.

### Generation of Mlkl knockout MVT-1 cells

Mouse mammary cancer cell line MVT-1 was cultured in DMEM containing 10% FBS, 2 mM l-glutamine, 100 U/ml penicillin and 100 mg/ml streptomycin. For targeting Mlkl with CRISPR/Cas9, lentiviral sgRNA vector targeting Mlkl was constructed by ligation of hybridized oligos into lentiCRISPR (pXPR-001, GeCKO) vector: Oligo1 (5′-caccgcgtctaggaaaccgtgtgca-3′) and Oligo2 (5′-aaactgcacacggtttcctagacgc-3′). The lentiCRISPR plasmid (with sgRNA cloned) and packaging plasmids pVSVg (Addgene, #8454) and psPAX2 (Addgene, #12260) were transfected into HEK293T cells for 48 h to generate lentivirus. MVT-1 cells were infected with the lentivirus for 24 h, followed by selection with puromycin (2 ug/ml, Life Technologies) for 5 days. Protein expression was determined by immunoblot analysis.

### Cell sorting and FACS

Peripheral blood cells were separated with Lympholyte-Mammal (CL5115, Cedarlane Labs). Tumor-infiltrating lymphocytes (TILs) were isolated through magnetic isolation followed by fluorescence activated cell sorting and FACS. Briefly, tumor tissue was dissociated with gentleMACS Dissociator to get single-cell suspension. Cells were blocked with anti-mouse CD16/32 antibody (Clone 93; BioLegend) and stained APC anti-mouse CD45 (Clone 30-F11; BioLegend). Subsequently, total CD45 + tumor-infiltrating leukocytes were magnetically isolated by incubating with anti-APC magnetic beads (Miltenyi Biotec). Leukocytes were then stained with DAPI (Thermo Fisher) plus PE anti-mouse CD3 antibody (Clone 17A2; BioLegend), PE/Cyanine7 anti-mouse CD4 antibody (clone GK1.5; BioLegend), BB515 Anti-Mouse CD8a (Clone 53-6.7; BD Horizon). CD4 + lymphocytes and CD8+ lymphocytes were sorted from total CD45 + cells by using a BD FACSAria Fusion sorter (BD Biosciences).

For FACS, cells were blocked with anti-mouse CD16/32 antibody and stained with Aqua Dead cell staining dye (Thermo Fisher) plus APC anti-mouse CD45 (Clone 30-F11; BioLegend), PE anti-mouse CD3 antibody (Clone 17A2; BioLegend), FITC anti-mouse CD4 antibody (clone GK1.5; BioLegend), Percp/Cyanine5.5 Anti-Mouse CD8a (Clone 53–6.7; BioLegend). Intracellular cytokines staining was performed using the Fixation/Permeabilization Solution Kit (BD Biosciences), Pacific Blue anti-Granzyme B Antibody (Clone GB11; BioLegend) and PE/Cyanine7 anti-mouse IFNγ antibody (Clone XMG1.2; BioLegend). Flow cytometry was carried out on FACSCanto™ II flow cytometer (BD Biosciences). Data were analyzed using FlowJo v.10.7.1 (Treestar, Ashland, OR). Gating strategies for cell sorting and FACS can be found in Extended Data Figure S2a.

### BMDMs active T cells with tumor antigen

Bone marrow–derived macrophages (BMDMs) were differentiated from mouse bone marrow cells with rmM-CSF (10 ng/ml, 416-ML-050/CF, R&D) for 7 days. LLC cells in culture medium (50 × 10^6^ cells/ml) were subjected to 5 rapid freeze–thaw cycles in dry ice and 55℃, and then centrifuged at 5000 rpm to collect supernatant.

BMDMs plated on 24-well plate (2 × 10^5^ cells/well) were treated with LLC cell lysate (12ul/well) for 18 h. Then, freshly isolated splenocytes (1 × 10^6^ cells/well) were added to the BMDMs in fresh DMEM medium with CD40L (0.5 ug/ml, 34-8512-80, Invitrogen) and IL-2 (50 U/ml, 575402, BioLegend). Additionally, the following was used as indicated: rmE-cadherin (10 ug/ml, 8875-EC-050, R&D), anti-E-Cadherin antibody (5 ug/ml, U5885, Sigma-Aldrich), Anti-KLRG1 antibody (5 ug/ml, 16-5893-85, Invitrogen). After 4 days, splenocytes were analyzed by FACS.

### Histology analysis

Tumors were bisected into two pieces in the middle of the tumor at the longest diameter orientation using a razor blade: One-half of the tumor was immediately placed in 4% buffered formalin (Z-fix) overnight, and the other half was frozen for protein extraction. The fixed tumors were embedded in paraffin and cut into 5-μm-thick serial sections staining using standard histological procedures. Every 3rd slide was routinely stained with hematoxylin and eosin as described [35]. Tumor sections from the center of excised tumors of similar size were used for analysis. Tumor necrosis was designated on H&E-stained slides as areas of dark-hematoxylin-stained necrotic tumor cells immediately adjacent to light-hematoxylin-stained viable tissues [36]. The quantitation of tumor necrotic/death area was counted using Image J and represented as the percentage of tumor necrotic/death area within whole tumor. Mice tumor sections (4 µm) were deparaffinized by incubation at 56 °C for 30 min and subsequent xylene washes then rehydrated with a graded ethanol. The paraffin sections were subjected to antigen retrieval with retrieval buffer (Dako) at 95 °C for 10 min and cooled down until room temperature. The slides were then treated with 3% H_2_O_2_ for 5 min washed with phosphate-buffered saline (PBS). The slides then were blocked with 2% normal goat serum, followed by overnight incubation with primary antibodies against p-MLKL (1:5000) or cl.Casp-3 (1:1000). Signals were developed using VECTASTIN ABC Elite kit (Vector Laboratories) and DAB Substrate Kit (Vector Laboratories) followed by manufacturer’s instructions. The slides were counter stained with Hematoxylin (Vector Laboratories) to detect nucleus. For the quantitation of metastatic burden, paraffin-embedded lung tissues were sectioned 400 μm apart throughout the whole lung followed by H&E staining. The frequency of the metastatic foci was counted manually in a blinded fashion.

### Western blot

Tumor tissues and in vitro cultured cells were lysed in RIPA buffer. Tumor lysates were separated by SDS-PAGE, followed by probing with anti-mouse MLKL antibody (Clone EPR17514; Abcam), anti-Actin antibody. Signals were developed by using enhanced chemiluminescence kit (Bio-Rad).

### Quantitative RT-PCR

The sorted T cells were subjected to RNA extraction using RNeasy Mini Kit (Qiagen). cDNA synthesis was conducted using PrimeScript RT reagent Kit (Roche, RR037A). Predesigned primers and probes for β-Actin, IFN-γ, T-bet (Integrated DNA Technologies) were used for qPCR Assays, and relative mRNA expression was measured using SensiFAST Probe Hi-ROX Mix (Bioline) on QuantStudio 3 Real-Time PCR System (Thermo Fisher Scientific). 2−ΔΔCT method was used to quantify fold induction, and each cDNA was normalized by β-Actin expression.

### ELISA

For serum samples, blood samples were clotted for 2 h at room temperature and centrifuged at 2000*g* for 20 min. For TIF, tumor tissue was put on 10 µm Nylon Net filter (NY1002500, Sigma), centrifuged at 60 g for 10 min and collect fluid. Samples were then analyzed for mouse JAM-A (ab277080, Abcam), mouse CD138 (NBP2-76610, Novus) and mouse E-cadherin (ab197751, Abcam) according to manufacturer’s instruction.

### Colony formation assay

The soft agar colony formation assay was performed as previously described [37]. 12-well plate was coated with a 1 ml base layer containing 1% agarose (Lonza 50111 SeaPlaque GTG Agarose). Peripheral blood was first depleted of red blood cells (RBCs) by ACK lysing buffer (118–156-101, Quality Biological). After washing and centrifugation, cell pellets were resuspended in 600ul DMEM containing 10% FBS, 2 mM l-glutamine, 100 U/ml penicillin, 100 mg/ml streptomycin and 0.5% agarose at 37 °C and added to 12-well plates. After solidified at room temperature, 500ul DMEM was added containing 10% FBS, 2 mM l-glutamine, 100 U/ml penicillin and 100 mg/ml streptomycin and cultured at 37 °C with 5% CO_2_ for 2 weeks. Colonies were stained with crystal violet and quantified.

### Statistical analysis

All data were analyzed with the GraphPad Prism 8 software. Student’s t test was used to determine the statistical significance of differences between groups. Differences with *p* values < 0.05 were considered significant.

## Results

### Inhibition of tumor necroptosis leads to the dramatic elevation of the anti-tumor activity of T cells

**Fig. 1:**
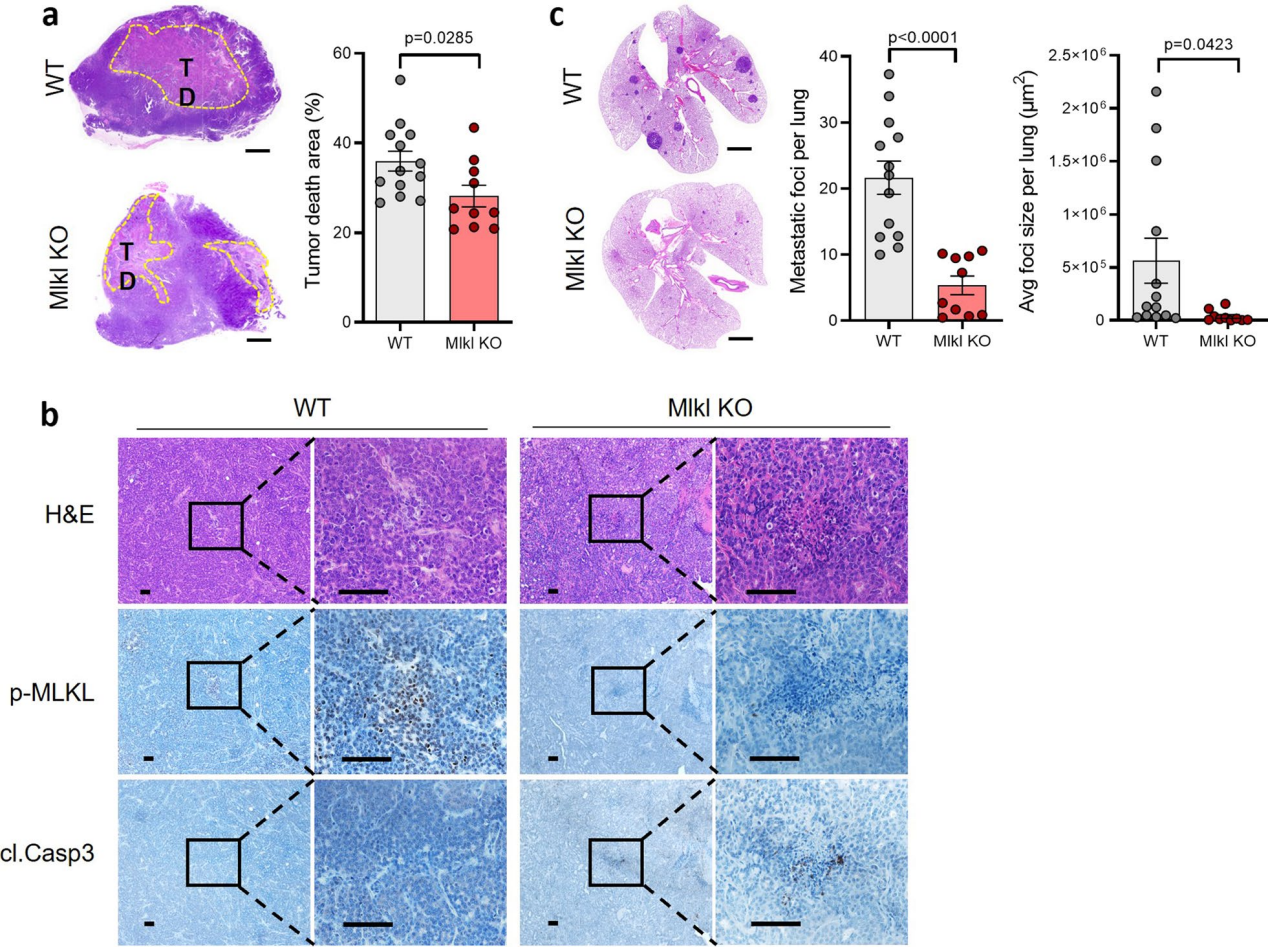
Necroptosis promotes lung metastasis. **a** Representative image of H&E-stained tumors (upper panel) from 15-week WT (*n* = 13) and Mlkl KO (*n* = 10) MMTV-PyMT mice. Percent of tumor death area (lower panel) of total tumor from mice at 15 weeks. Scale bar, 2 mm. **b** Representative images of H&E and immunohistological stained tumor sections from 15-week WT (*n* = 13) and Mlkl KO (*n* = 10) MMTV-PyMT mice as in Fig. a, stained with phospho-MLKL (p-MLKL) or cleaved caspase-3 (cl.Casp-3) antibodies. Scale bar, 50 μm. Experiments were repeated three times. **c** Left panel shows the representative images of H&E-stained lung sections from 15-week WT (*n* = 13) and Mlkl KO (*n* = 10) MMTV-PyMT mice as in Fig. a. Scale bar, 2 mm. Middle panel shows the quantification of metastatic foci in lungs from 15-week WT (*n* = 13) and Mlkl KO (*n* = 10) MMTV-PyMT mice. Right panel shows the average size of metastatic foci in lungs. Data presented as the mean ± sem and *p* value was determined by two-tailed t test (**a**, **c**)

In our recent studies, we found that blocking necroptosis in tumor cells resulted in the inhibition of lung metastasis in the orthotopic transplantation breast cancer MVT-1 model [19, 30]. To confirm the role of tumor necroptosis in breast cancer metastasis, we examined the effect of MLKL deletion on lung metastasis in the genetically engineered MMTV-PyMT spontaneous breast cancer model. We first generated Mlkl knockout (KO) MMTV-PyMT mice by Cas9/CRISPR-mediated genome editing (Additional file 1: Fig. S1a, b). The expression of MLKL showed tissue-specific pattern in liver, lung, spleen, and intestine but not in heart or kidney, and MLKL protein expression in these tissues was completely absent in Mlkl KO mice (Additional file 1: Fig. S1c). We then crossed these female Mlkl heterozygous mice with male MMTV-PyMT Mlkl heterozygous mice to generate Mlkl KO and wild-type (WT) MMTV-PyMT littermates for examining tumor growth and lung metastasis. In our previous study, we found that MLKL expression in normal breast tissue is low but highly increased in MMTV-PyMT tumors [30]. We confirmed that MLKL expression was also completely abolished in Mlkl KO MMTV-PyMT tumors (Additional file 1: Fig. S1d). The primary tumor growth in WT and Mlkl KO MMTV-PyMT mice was similar (Additional file 1: Fig. S1e, f). When we checked the tumor death areas in tumors of WT and Mlkl KO MMTV-PyMT mice, Mlkl KO tumors showed less tumor death area at 15 weeks (Fig. 1a). We next examined the levels of phosphorylated MLKL and cleaved caspase-3 in the death areas of both WT and KO tumors. As expected, p-MLKL positive cells were only detected in the death areas of WT, but not Mlkl KO tumors (Fig. 1b, middle panel). Conversely, the cleaved Casp-3 staining was mostly observed in Mlkl KO tumors (Fig. 1b, bottom panel), indicating that apoptosis is significantly increased in Mlkl KO tumors. Importantly, we found that lung metastasis was significantly decreased in Mlkl KO MMTV-PyMT mice and specifically, and the average foci size of metastasized tumors in the lung was dramatically reduced in Mlkl KO MMTV-PyMT tumors (Fig. 1c). These results are consistent with our previous findings in the MVT-1 transplantation model [30] and confirmed that necroptosis is the predominant form of cell death in tumor death areas, blocking necroptosis switches tumor necroptosis to tumor apoptosis, and most importantly, inhibition of tumor necroptosis leads to the reduced lung metastasis.

**Fig. 2:**
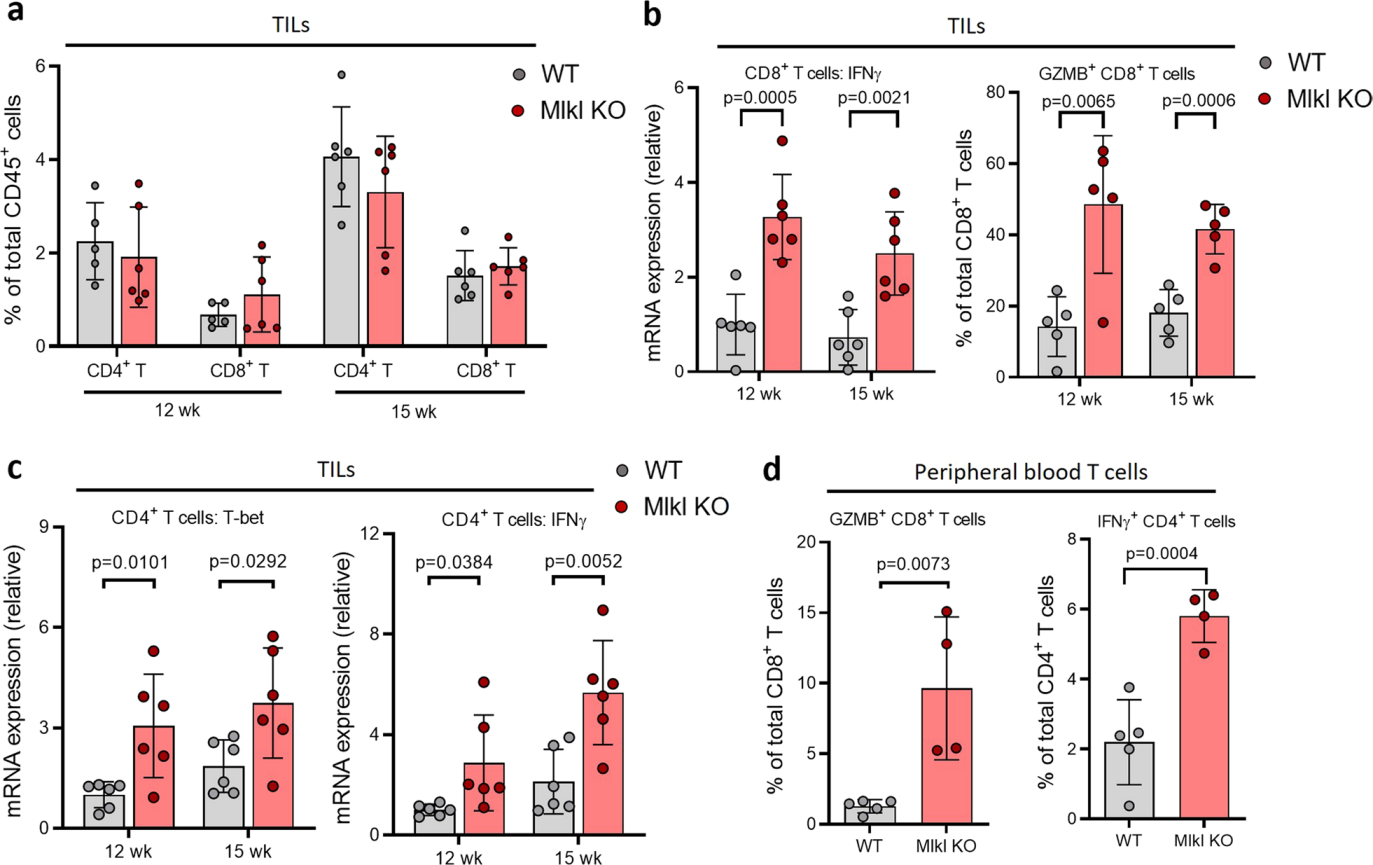
Necroptosis of tumor cells promotes T cell activation. **a** Flow cytometry analysis of tumor-infiltrating CD4+ T and CD8+ T cells in WT (*n* = 5), Mlkl KO (*n* = 6) MMTV-PyMT mice at 12 weeks and WT (*n* = 6), Mlkl KO (*n* = 6) MMTV-PyMT mice at 15 weeks. No significant difference in *p* value was observed. **b** qPCR analysis of IFN-γ mRNA expression in tumor-infiltrating CD8+ T cells of WT and Mlkl KO MMTV-PyMT mice at 12 weeks and WT (*n* = 6) and 15 weeks (*n* = 5, each) (left panel). Flow cytometry analysis of tumor-infiltrating GZMB+ CD8+ T cells in WT and Mlkl KO MMTV-PyMT mice at 12 weeks and WT (*n* = 6) and 15 weeks (*n* = 5, each) (right panel). **c** qPCR analysis of T-bet and IFN-γ mRNA expression in tumor-infiltrating CD4+ T cells of WT and Mlkl KO MMTV-PyMT mice at 12 weeks and 15 weeks (*n* = 6, each). **d** Flow cytometry analysis of peripheral blood GZMB+ CD8+ T cells and IFN-γ+ CD4+ T cells in WT (*n* = 5) and Mlkl KO (*n* = 4) MMTV-PyMT mice at 15 weeks. Data presented as the mean ± sem and *p* value was determined by two-tailed t test (**a**–**d**)

Since it has been reported that necroptosis may have a suppressing or promoting effect on tumor growth through modulating tumor immunity [38–40] and that the inhibition of the tumor-suppressing functions of T cells plays a key role in tumor metastasis [31, 32], we then explored if the tumor-suppressing function of T cells is affected by tumor necroptosis in both MMTV-PyMT and MVT-1 models. We first checked if the deletion of MLKL has any effect on T cell infiltration in Mlkl KO tumors and found that MLKL deletion did not significantly affect the total populations of CD4^+^ and CD8^+^ T cells in the tumors of MMVT-PyMT model (Fig. 2a, Additional file 1: Fig. S2a). As tumor necroptosis normally starts to happen in tumors at around 10 weeks in the MMTV-PyMT breast tumor model [30], we then collected tumors at 10, 12 and 15 weeks from WT and Mlkl KO MMTV-PyMT mice and examined the infiltrating T cell anti-tumor activity by measuring the levels of IFN-γ and the percentage of Granzyme B positive cells, two markers of CD8^+^ T cell functions and the levels of T-bet and IFN-γ (Th1) of CD4^+^ T cells [41]. While there is no difference of T cell activity between 10-week WT and Mlkl KO tumors (Additional file 1: Fig. S2b), we found that the levels of IFN-γ and the percentage of Granzyme B positive cells were dramatically elevated in the CD8^+^ T cells of Mlkl KO MMTV-PyMT tumors (Fig. 2b) and that CD4^+^ T cells of Mlkl KO tumors exhibited significant increase in the expression of T-bet (Th1) and IFN-γ (Th1) mRNA (Fig. 2c). These results suggest that inhibition of necroptosis elevates both cytotoxic and Th1 activity of infiltrating T cells in MMVT-PyMT tumors. To find out if tumor necroptosis has any effect on the anti-tumor activity of peripheral T cells, we also examined the T cells isolated from the blood and spleens of WT and Mlkl KO MMVT-PyMT mice. Interestingly, we found that the anti-tumor activity of the circulating T cells from blood of Mlkl KO mice was also dramatically increased compared to the cells of WT mice; however, the activity of T cells from spleens is quite similar in WT and Mlkl KO mice (Fig. 2d; Additional file 1: Fig. S2c). These results indicated that necroptosis inhibition also leads to the increase in the anti-tumor activity of the circulating T cells. We obtained similar results of the activity of tumor-infiltrating and peripheral T cells from the MVT-1 model (Additional file 1: Fig. S2d-h). Taken together, the above findings suggest that necroptosis may promote tumor metastasis through inhibiting T cell anti-tumor activity.

### Deletion of CD8+ T cells restores tumor metastasis in mice bearing Mlkl KO tumors

**Fig. 3:**
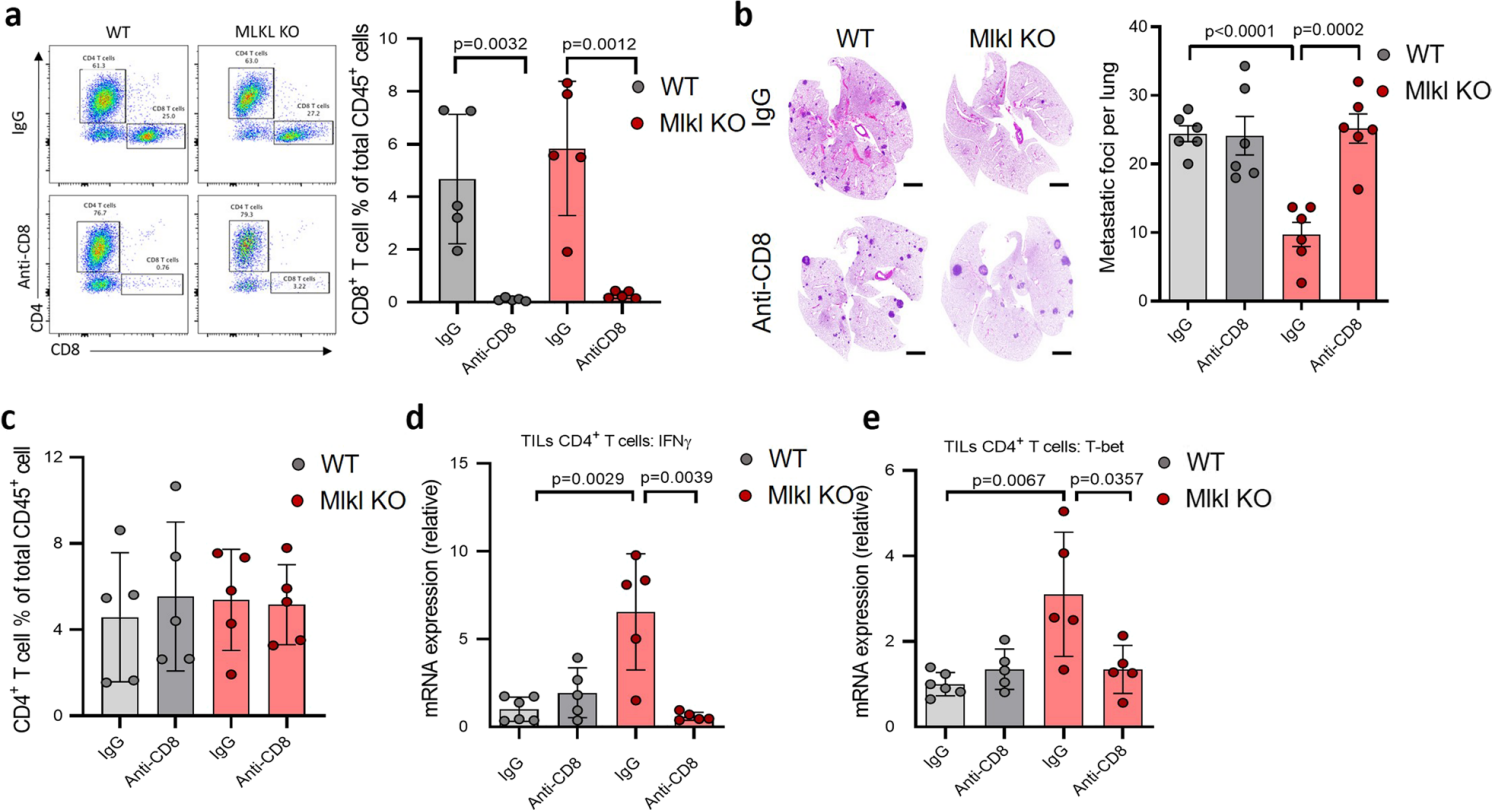
CD8+ T cells play important role in necroptosis-mediated tumor metastasis. **a** Right panel, flow cytometry gating strategy for tumor-infiltrating CD8+ T cells. Left panel, flow cytometry analysis of tumor-infiltrating CD8+ T cells in WT and Mlkl KO MMTV-PyMT mice treated with IgG or anti-CD8 antibody (*n* = 5, each). **b** Left panel shows the representative images of H&E-stained lung sections from WT and Mlkl KO MMTV-PyMT mice treated with IgG or anti-CD8 antibody. Scale bar, 2 mm. Right panel shows the quantification of metastatic foci in lungs (*n* = 6, each). **c** Flow cytometry analysis of tumor-infiltrating CD4+ T cells in WT and Mlkl KO MMTV-PyMT mice treated with IgG or anti-CD8 antibody (*n* = 5, each). No significant difference in *p* value was observed. **d** and **e**, qPCR analysis of IFN-γ and T-bet mRNA expression of tumor-infiltrating CD4+ T cells in WT and Mlkl KO MMTV-PyMT mice treated with IgG or anti-CD8 antibody (*n* = 5, each). Data presented as the mean ± sem and *p* value was determined by two-tailed t test (**a**–**e**)

To make sure that the inhibition of T cell anti-tumor activity by necroptosis is responsible for the promoting effect of necroptosis on tumor metastasis, we tested if the deletion of CD8+ T cells could restore tumor metastasis in mice bearing Mlkl KO tumors in both models. To do so, CD8+ T cells were depleted from mice with WT or Mlkl KO tumors in both MMTV-PyMT and MVT-1 models with an anti-CD8 antibody (Fig. 3a, Additional file 1: Fig. S3a). While the depletion of CD8+ T cells had no effect on lung metastasis of the WT MMTV-PyMT and MVT-1-CRISPR-CT tumors, however, the loss of CD8+ T cells in mice with Mlkl KO MMTV-PyMT and Mlkl KO MVT-1 tumors leads to the restoration of the metastasis of breast tumor cells to the lung almost as efficiently as their WT counterparts (Fig. 3b and Additional file 1: Fig. S3b). To examine the effect of CD8+ T cell depletion on CD4+ T cells, we analyzed the expression levels of T-bet and IFN-γ in tumor-infiltrating CD4+ T cells. Although CD8+ T cell depletion did not alter the CD4+ T cell populations in both WT and KO tumors, the increased expression levels of Th1-related genes T-bet and IFN-γ in the CD4+ T cells of Mlkl KO tumors were abolished in the absence of CD8+ T cells (Fig. 3c–e, Additional file 1: Fig. S3c, d). These results indicated that the increased anti-tumor activity of CD8+ T cells upon necroptosis blockage plays a pivotal role in modulating tumor immunity and lung metastasis.

### Necroptosis-mediated ectodomain shedding of cell surface proteins promotes tumor metastasis

**Fig. 4:**
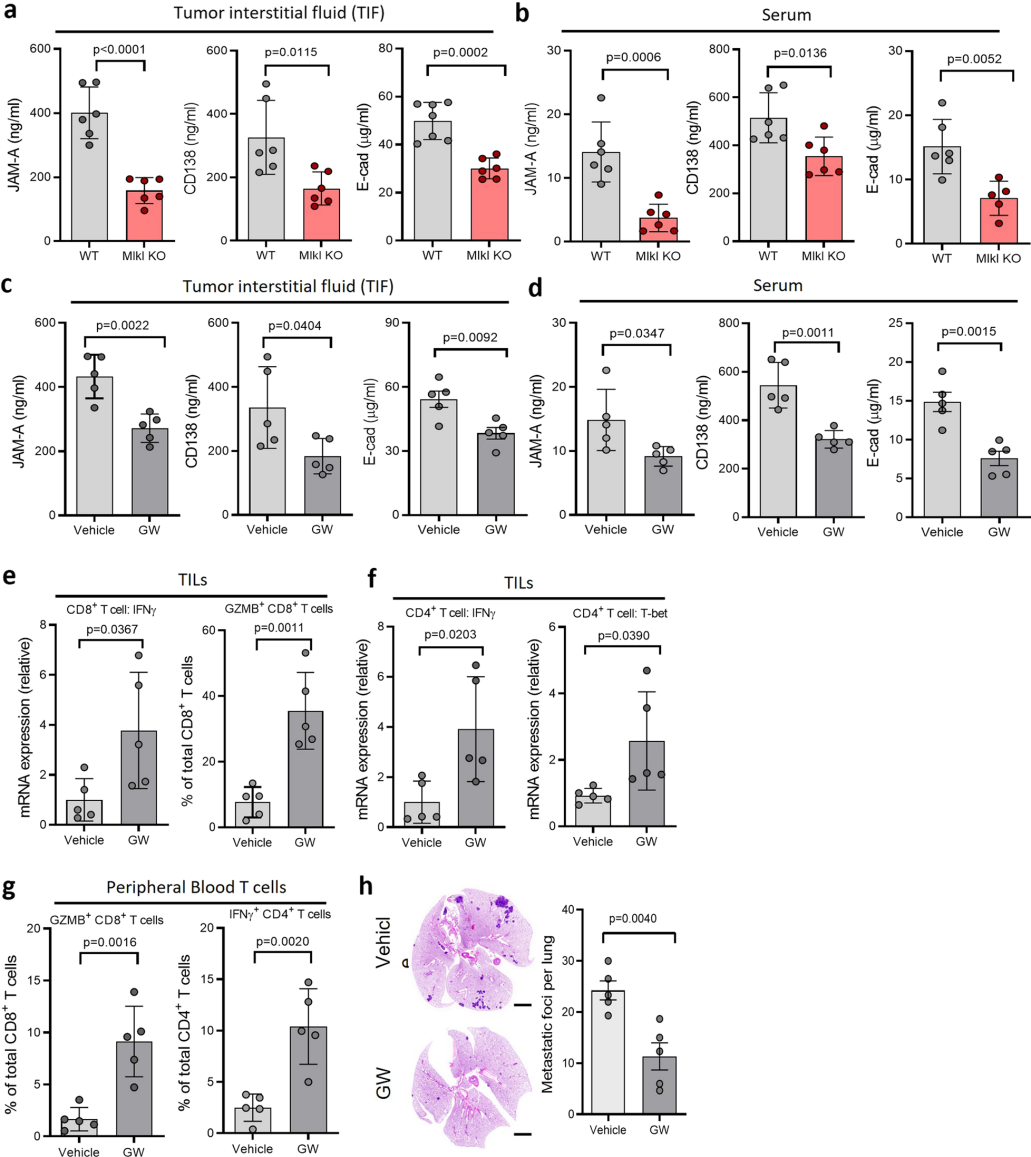
Necroptosis promotes tumor metastasis by activating cell surface proteases. **a**, **b** ELISA of soluble JAM-A, CD138 and E-cad in TIF(**a**) and serum (**b**) from WT and Mlkl KO MMTV-PyMT mice at 15 weeks (*n* = 6, each). **c**, **d** ELISA of soluble JAM-A, CD138 and E-cad in TIF (**c**) and serum (**d**) from MMTV-PyMT mice treated with vehicle or GW280264X (*n* = 5, each). **e** qPCR analysis of IFN-γ mRNA expression in tumor-infiltrating CD8+ T cells of MMTV-PyMT mice treated with vehicle or GW280264X (*n* = 5, each; left panel). Flow cytometry analysis of tumor-infiltrating GZMB+ CD8+ T cells in MMTV-PyMT mice treated with vehicle or GW280264X (*n* = 5, each; right panel). **f** qPCR analysis of IFN-γ and T-bet mRNA expression in tumor-infiltrating CD4+ T cells of MMTV-PyMT mice treated with vehicle or GW280264X (*n* = 5, each). **g** Flow cytometry analysis of peripheral blood GZMB+ CD8+ T cells and IFN-γ+ CD4+ T cells MMTV-PyMT mice treated with vehicle or GW280264X (*n* = 5, each). **h** Left panel shows the representative images of H&E-stained lung sections from MMTV-PyMT mice treated with vehicle or GW280264X.Scale bar, 2 mm. Right panel shows the quantification of metastatic foci in lungs from MMTV-PyMT mice treated with vehicle or GW280264X (*n* = 5, each). Data presented as the mean ± sem and *p* value was determined by two-tailed t test (**a**–**h**)

As our previous study showed that necroptosis leads to MLKL-mediated activation of the cell surface protease, ADAMs and the ectodomain shedding of cell surface proteins [21]. It is also reported that several soluble cell surface proteins including E-cadherin (sE-cad), junctional adhesion molecule A (JAM-A) and Syndecan-1 (CD138) promote breast tumor metastasis [22–24]. We then examined if these soluble cell surface proteins exist in the tumor interstitial fluid (TIF) of 15-week MMTV-PyMT tumors and in the serum of tumor bearing mice. While significant amounts of these three soluble proteins were found in the TIF of WT MMTV-PyMT tumors and in the serum of mice bearing WT tumors, the levels of these proteins are substantially lower in both TIF and the serum from Mlkl KO tumors and mice bearing Mlkl KO tumors (Fig. 4a, b). Similar results were seen in the TIF and the serum from WT and Mlkl KO MVT-1 tumors and the tumor bearing mice (Additional file 1: Fig. S4a, b). To confirm that the elevated levels of these three soluble proteins are resulted from necroptosis-mediated shedding of their cell surface-anchored full-length proteins, we treated 12-week WT MMTV-PyMT mice with either vehicle or GW280264X (GW), a ADAM10/ADAM17 inhibitor, once every 3 days for three weeks as ADAM10 and ADAM17 are the predominant variants of ADAMs expressed in mouse cells based on our previous study [21]. We then examined the levels of these three soluble proteins in TIF and serum, the anti-tumor activity of T cells and lung metastasis of these mice at 15 weeks. First, there was marked decrease in the levels of JAM-A, CD138 and sE-cad in the TIF and serum of GW-treated mice (Fig. 4c, d). These results suggest that the elevated levels of these three soluble proteins of WT MMTV-PyMT mice are dependent on ADAM activity. Next, we examined the activation of T cells and found that the anti-tumor activity of both tumor-infiltrating and peripheral blood T cells was significantly up-regulated in mice treated with GW (Fig. 4e–g). The activity of spleen T cells was slightly increased in GW-treated mice (Additional file 1: Fig. S4c, d). Finally, while GW treatment had little effect on primary tumor growth and the levels of necroptosis in tumors as compared to that of vehicle-treated mice (Additional file 1: Fig. S4e, f), we found that GW-treated mice had significantly reduced lung metastasis as compared to the control mice (Fig. 4h). These data suggest that the necroptosis-mediated shedding of cell surface proteins is critical to promote metastasis.

### Necroptosis suppresses the anti-tumor activity of T cells through the co-inhibitory receptor KLRG1

**Fig. 5:**
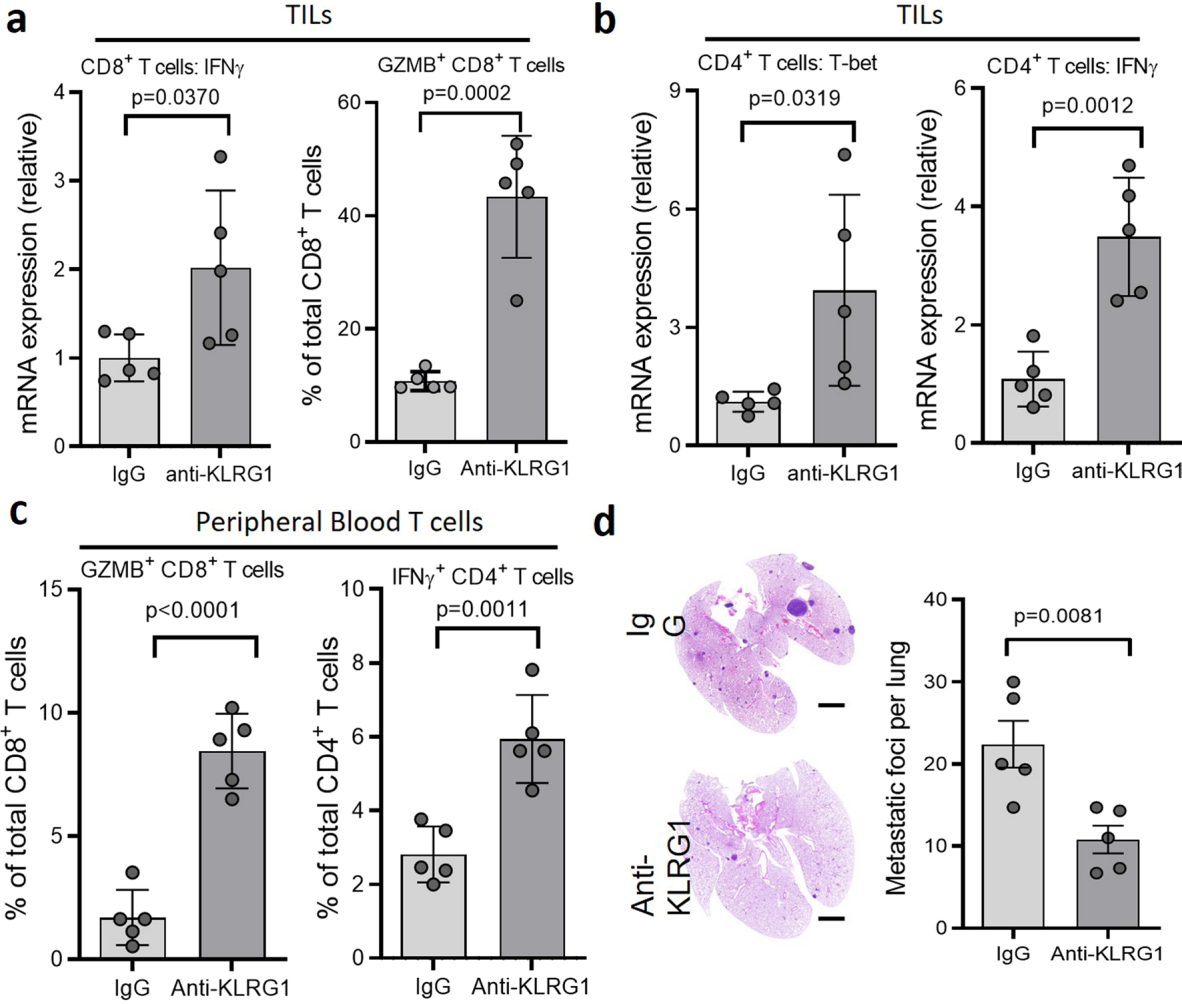
Blocking E-cad/KLRG1 pathway inhibits tumor metastasis. **a** qPCR analysis of IFN-γ mRNA expression in tumor-infiltrating CD8+ T cells of MMTV-PyMT mice treated with IgG or Anti-KLRG1 antibody (*n* = 5, each; left panel). Flow cytometry analysis of tumor-infiltrating GZMB+ CD8+ T cells in MMTV-PyMT mice treated with IgG or Anti-KLRG1 antibody (*n* = 5, each; right panel). **b** qPCR analysis of T-bet and IFN-γ mRNA expression in tumor-infiltrating CD4+ T cells of MMTV-PyMT mice treated with IgG or Anti-KLRG1 antibody (*n* = 5, each). **c** Flow cytometry analysis of peripheral blood GZMB+ CD8+ T cells and IFN-γ+ CD4+ T cells MMTV-PyMT mice treated with IgG or Anti-KLRG1 antibody (*n* = 5, each). **d** Left panel shows the representative images of H&E-stained lung sections from MMTV-PyMT mice treated with IgG or Anti-KLRG1. Scale bar, 2 mm. Right panel shows the quantification of metastatic foci in lungs from MMTV-PyMT mice treated with IgG or Anti-KLRG1 (*n* = 5, each). Data presented as the mean ± sem and *p* value was determined by two-tailed t test (**a**–**d**)

Killer cell lectin-like receptor G1, KLRG1, is a co-inhibitory receptor and expressed on antigen-experienced T cells and NK cells [42]. KLRG1 is known as a marker of T cell senescence. Recent reports suggest that it plays an inhibitory role in regulating T cell functions [43, 44]. As sE-cad has been reported to be a ligand for KLRG1 and we observed significant elevation of sE-cad levels in TIF and serum of WT tumors and mice, we then investigated the possibility that necroptosis engages KLRG1 to inhibit the anti-tumor activity of T cells. We first examined KLRG1 expression in T cells of WT and Mlkl KO MMTV-PyMT mice and found that the population of KLRG1 expressing T cells in tumor-infiltrating and spleen T cells are not affected by MLKL deletion (Additional file 1: Fig. S5a). After confirming that sE-cad could inhibit T cell activation using the in vitro assay of tumor/macrophage-mediated CD8+ T cell activation (Additional file 1: Fig. S5b), we tested if a neutralizing anti-KLRG1 antibody could reverse the inhibitory effect of sE-cad on T cell activation and found that the anti-KLRG1 antibody was able to significantly reduce the inhibition of T cell activation by sE-cad (Additional file 1: Fig. S5c). To test if blocking KLRG1 engagement with this neutralizing antibody has any effect on the anti-tumor activity of T cells in the MMTV-PyMT model, we treated 12-week WT MMTV-PyMT mice with either isotype control or anti-KLRG1 antibody once every 3 days for three weeks. The anti-tumor activity of T cells of these mice was evaluated at 15 weeks. The anti-KLRG1 antibody treatment had little affect compared to mice treated with the control isotype antibody (Additional file 1: Fig. S5d). The anti-tumor activity of tumor-infiltrating T cells and peripheral blood T cells is dramatically up-regulated in mice treated with the anti-KLRG1 antibody (Fig. 5a–c). Interestingly, the anti-tumor activity of the spleen T cells is also increased following anti-KLRG1 treatment (Additional file 1: Fig. Se, f). Importantly, blocking KLRG1 engagement significantly reduced lung metastasis (Fig. 5d). These results suggest that the sE-cad/KLRG1 pathway plays a critical role in necroptosis-triggered dampening of the anti-tumor activity of T cells and in promoting metastasis in MMTV-PyMT breast cancer model.

As some recent reports suggested that PD-L1/PD-1 pathway may play a role in controlling the development of certain types of breast cancer [45, 46], we examined the levels of PD-L1 in TIFs and serum and PD-1 levels in T cells of WT, Mlkl KO or GW-treated WT MMTV-PyMT mice and found that while there are low levels of soluble PD-L1 in TIFs, not in serum, the amounts of PD-L1 in these mice are quite similar regardless of MLKL status or GW treatment (Additional file 1: Fig. S5g). Also, the PD-1 levels in T cells of WT and Mlkl KO mice are similar (Additional file 1: Fig. S5h). Therefore, it is unlikely that PD-L1/PD-1 pathway plays a major role in necroptosis-triggered promoting effect on metastasis. Hence, KLRG1 is likely the major inhibitory receptor engaged by necroptosis to inhibit the anti-tumor activity of T cells in MMTV-PyMT breast cancer model.

## Discussion

Tumor metastasis is the main cause of cancer mortality and is a complex process modulated by many different factors including the interplays between tumor cells and host immunity [47, 48]. Particularly, host T cells play a critical role in controlling metastasis and can exert their anti-tumor activity in tumors as tumor-infiltrating T cells or attack circulating tumor cells peripherally as peripheral T cells [31]. Inhibiting the anti-tumor activity of T cells is a major feature of the interplay of tumor cells and host immunity during tumor development and metastasis [31, 32]. In the current study, we found that the ectodomain shedding of cell surface proteins of necroptotic tumor cells leads to the accumulation of soluble cell surface proteins in tumor microenvironments, resulting in the inhibition of the anti-tumor activity of tumor-infiltrating and peripheral T cells and the promotion of tumor metastasis.

Although evading apoptosis is one of the hallmarks of cancer, tumor cell death happens during tumor development. Foci of cell death, referred as tumor necrosis, are commonly observed in core regions of solid tumors as a result of inadequate vascularization and subsequent metabolic stresses such as hypoxia and nutrient deprivation [49, 50]. Tumor necrosis is often associated with aggressive tumor development and metastasis [51, 52]. Unlike apoptosis in which cells have intact membranes and are rapidly removed by host macrophages, tumor necrosis often results in the release of cellular components to the tumor microenvironment and may lead to the alteration of the tumor microenvironment [17, 53]. We recently demonstrated that necroptosis is the major form of cell death that causes tumor necrosis in MVT-1 breast cancer and blocking of necroptosis dramatically abolished lung metastasis [19, 30]. On the other hand, apoptosis is accounted for the remaining tumor cell death in MLKL KO tumors [19, 30]. We found that both intrinsic and extrinsic apoptotic pathways are engaged in MLKL KO tumors as both cleaved Casp-8 and Casp-9 are detected (Additional file 1: Fig. S5i). Some papers show that pyroptosis also involves in the alteration of the tumor microenvironment [54]. However, we did not observe pyroptosis in MMTV-PyMT tumors (Additional file 1: Fig. S5i).

In the current study with the genetically modified MMTV-PyMT breast cancer model, we have confirmed that blocking necroptosis by deletion of the key necroptosis effector protein, MLKL, dramatically inhibits lung metastasis, but has no or little effect on tumor initiation and growth at the stages of tumor development before necroptosis occurs. More importantly, we found that blocking necroptosis significantly elevated the anti-tumor activity of both tumor-infiltrating and peripheral blood T cells in both MMTV-PyMT and MVT-1 breast cancer models. Our findings suggest that necroptosis has a suppressive effect on the anti-tumor activity of T cells in breast cancer. Additionally, necroptosis may promote the survival of circulating tumor cells (CTCs) through inhibiting the activity of peripheral blood T cells in MVT-1 breast cancer model (Additional file 1: Fig. S5j).

While recent studies reported that necroptosis plays an important role in regulating tumor immunity [38–40], the exact role of necroptosis in tumor development may need to be further evaluated in different types of tumors and under different conditions, as current studies suggest that chronic and spontaneous necroptosis may promote tumor development due to its suppressive effect on anti-tumor immunity in certain types of cancer and that acute and massive induction of necroptosis by chemotherapy or radiation treatment may undermine tumor growth and triggers immunogenic responses [55]. However, the underlying mechanisms of necroptosis on tumor immunity remain elusive [56, 57]. Recently, we showed that necroptosis triggers the activation of cell surface proteases, ADAMs, which lead to the shedding and the release of cell surface proteins to become soluble proteins [21]. As several of the soluble proteins, sE-cad, JAM-A and CD138, are known to promote metastasis [22–24], we examined the levels of these proteins in TIFs and serum from WT and Mlkl KO tumors or mice and found that there is a marked increase in the levels of these proteins in both TIFs and serum from WT tumors or mice bearing WT tumors. Particularly, administration of ADAM inhibitor, GW280264X, dramatically reduced the levels of these molecules in MMTV-PyMT WT mice. More importantly, GW280264X led to the elevated anti-tumor activity of both tumor-infiltrating and peripheral T cells and the marked reduction of lung metastasis (Fig. 4). These findings suggest that necroptosis-mediated shedding of the surface proteins of tumor cells play a key role in inhibiting the anti-tumor activity of T cells and promoting metastasis.

Previous reports suggest that one of these proteins, sE-cad, functions as a ligand for the co-inhibitory receptor, KLRG1 [29]. As the well-known co-inhibitory factor of immune response, PD-1, does in regulating T cell activity, KLRG1 also plays a role in keeping T cell activation in check [43, 44]. While the expression levels of KLRG1 and PD-1 in T cells are normally increased during viral, bacterial or parasite infections [58–60], we found that the levels of KLRG1 and PD-1 in T cells remain the same regardless of MLKL status in MMTV-PyMT model (Extended Data. Figure 5). However, as the ligands of KLRG1 or PD-1, respectively, we found that the level of sE-cad, not PD-L1, is significantly increased in TIFs and serum of WT tumors or mice of MMTV-PyMT model (Fig. 5). Importantly, neutralizing KLRG1 with antibody significantly elevated the anti-tumor activity of tumor-infiltrating and peripheral T cells and dramatically reduced lung metastasis in MMTV-PyMT model (Fig. 5). Because the effect of KLRG1 neutralization on T cell activity and lung metastasis is comparable to that as due to MLKL deletion or ADAM inhibition, KLRG1, not the PD-1, pathway likely plays a major role in necroptosis-mediated inhibition of T cell anti-tumor activity and the promoting effect on metastasis.

## Conclusions

Our current study reveals a novel mechanism of necroptosis-mediated promotion of metastasis and provides compelling evidence for targeting ADAM proteases or necroptosis as a promising therapy in controlling metastasis of breast cancer.

## Supplementary Information

**Additional file 1**. Supplementary figures.

## Data Availability

The data generated in this study are available upon request from the corresponding author.

## Abbreviations

RIPK3: Receptor-interacting protein kinase
MLKL: Mixed lineage kinase domain-like protein
ADAMs: A disintegrin and metalloproteases
cIAPs: Cellular inhibitor of apoptosis proteins
ZBP1: Z-DNA-binding protein 1
EpCAM: Epithelial cellular adhesion molecule
sE-Cad: Soluble E-cadherin
JAM-A: Junctional adhesion molecule A
KLRG1: Killer cell lectin-like receptor subfamily G member 1
WT: Wild type
KO: Knockout
TIF: Tumor interstitial fluid
TIL: Tumor-infiltrating lymphocyte
