# Supplementary material for: Tumor necroptosis-mediated shedding of cell surface proteins promotes metastasis of breast cancer by suppressing anti-tumor immunity
Source: Breast Cancer Res. 2023 Jan 26;25:10. doi: 10.1186/s13058-023-01604-9 (PMC9881343; doi:10.1186/s13058-023-01604-9)
Supplement: Supplementary file 1 — Additional file 1. Supplementary figures. [file 13058_2023_1604_MOESM1_ESM.docx]

**Supplementary Figure S1.**

**
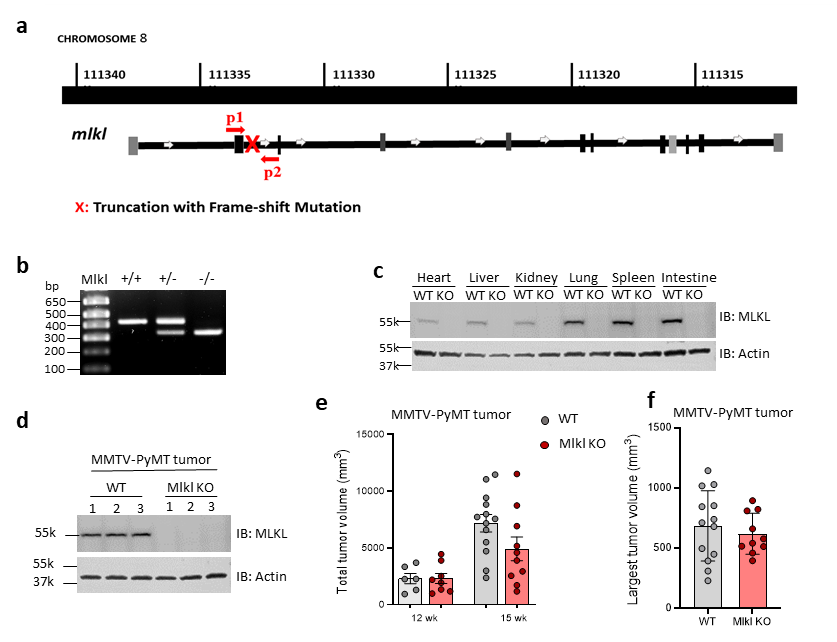
**

**Supplementary Figure S1. Loss of Mlkl does not affect primary tumor growth**. **a**, Mlkl gene scheme. a Mlkl knockout mouse was generated by targeting exon 2. An allele containing a 73-bp frame-shift deletion in exon 2 was chosen as the Mlkl-knockout allele. Locations of probe are indicated. **b**, PCR analysis of genomic DNA from mice, a single band at 437 bp indicating WT (+/+), a single 364 bp band indicating homozygous ko (-/-), and both bands, heterozygous (+/-). **c**, Immunoblot analysis of MLKL expression in various tissues from WT and Mlkl KO mice. **d**, Immunoblot analysis of MLKL in tumor lysates from WT and Mlkl KO mice. Each group contain 3 mice samples. **e**, Total tumor volume in WT (n=6) and Mlkl KO (n=8) MMTV-PyMT mice at 12 weeks and WT (n=13) and Mlkl KO (n=10) MMTV-PyMT mice at 15 weeks. **f**, The volume of the largest tumor from WT (n=13) and Mlkl KO (n=10) MMTV-PyMT mice at 15 weeks. Experiments were repeated more than three times, and representative data are shown (**b**-**d**). Data presented as the mean ± sem and p value was determined by two-tailed t-test (**e-f**).

**Supplementary Figure S2.**

**
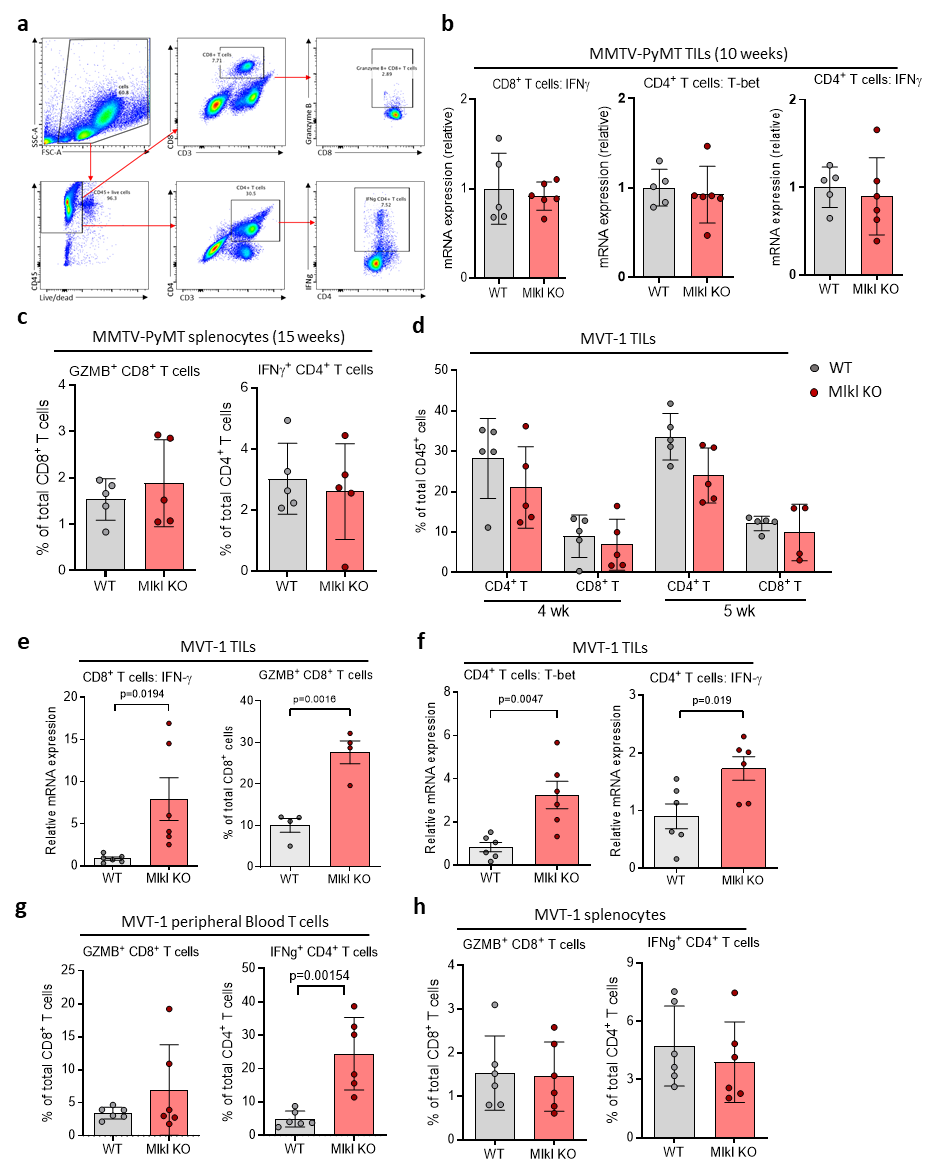
**

**Supplementary Figure S2. Necroptosis of tumor cells promotes T cell activation in MVT-1 tumor. a**, Flow cytometry gating strategy for GZMB+ CD8+ T cells and IFN-γ+ CD4+ T cells. **b**, qPCR analysis of IFN-γ and T-bet mRNA expression of tumor-infiltrating lymphocytes from WT (n=5) and Mlkl KO (n=6) MMTV-PyMT mice at 10 weeks. **c**, Flow cytometry analysis of spleen IFN-γ+ CD4+ T cells and GZMB+ CD8+ T cells in WT and Mlkl KO MMTV-PyMT mice at 15 weeks (n=5, each). No significant difference in p value was observed. **d**, Flow cytometry analysis of Tumor-infiltrating CD4+ T cells and CD8+ T cells from mice at 4- and 5-weeks post-implantation with MVT-1-sgCT (n=5, each) and MVT-1-Mlkl KO (n=5, each). No significant difference in p value was observed. **e**, qPCR analysis of IFN-γ mRNA expression of tumor-infiltrating CD8+ T from mice at 5 weeks post-implantation with MVT-1-sgCT and MVT-1-Mlkl KO cells (n=6, each; left panel). Flow cytometry analysis of tumor-infiltrating GZMB+ CD8+ T cells from mice at 5 weeks post-implantation with MVT-1-sgCT and MVT-1-Mlkl KO cells (n=4, each; right panel). **f**, qPCR analysis of T-bet and IFN-γ mRNA expression of tumor-infiltrating CD4+ T cells from mice at 5 weeks post-implantation with MVT-1-sgCT and MVT-1-Mlkl KO cells (n=6, each). **g**, Flow cytometry analysis of peripheral blood GZMB+ CD8+ T cells from mice at 5 weeks post-implantation with MVT-1-sgCT and MVT-1-Mlkl KO cells (n=6, each; left panel). Flow cytometry analysis of peripheral blood IFN-γ+ CD4+ T cells from mice at 5 weeks post-implantation with MVT-1-sgCT and MVT-1-Mlkl KO cells (n=6, each; right panel). **h**, Flow cytometry analysis of spleen GZMB+ CD8+ T cells from mice at 5 weeks post-implantation with MVT-1-sgCT and MVT-1-Mlkl KO cells (n=6, each; left panel). Flow cytometry analysis of spleen IFN-γ+ CD4+ T cells from mice at 5 weeks post-implantation with MVT-1-sgCT and MVT-1-Mlkl KO cells (n=6, each; right panel). No significant difference in p value was observed. Experiments were repeated more than three times, and representative data are shown (**a**). Data presented as the mean ± sem and p value was determined by two-tailed t-test (**b**-**h**).

**Supplementary Figure S3.**

**
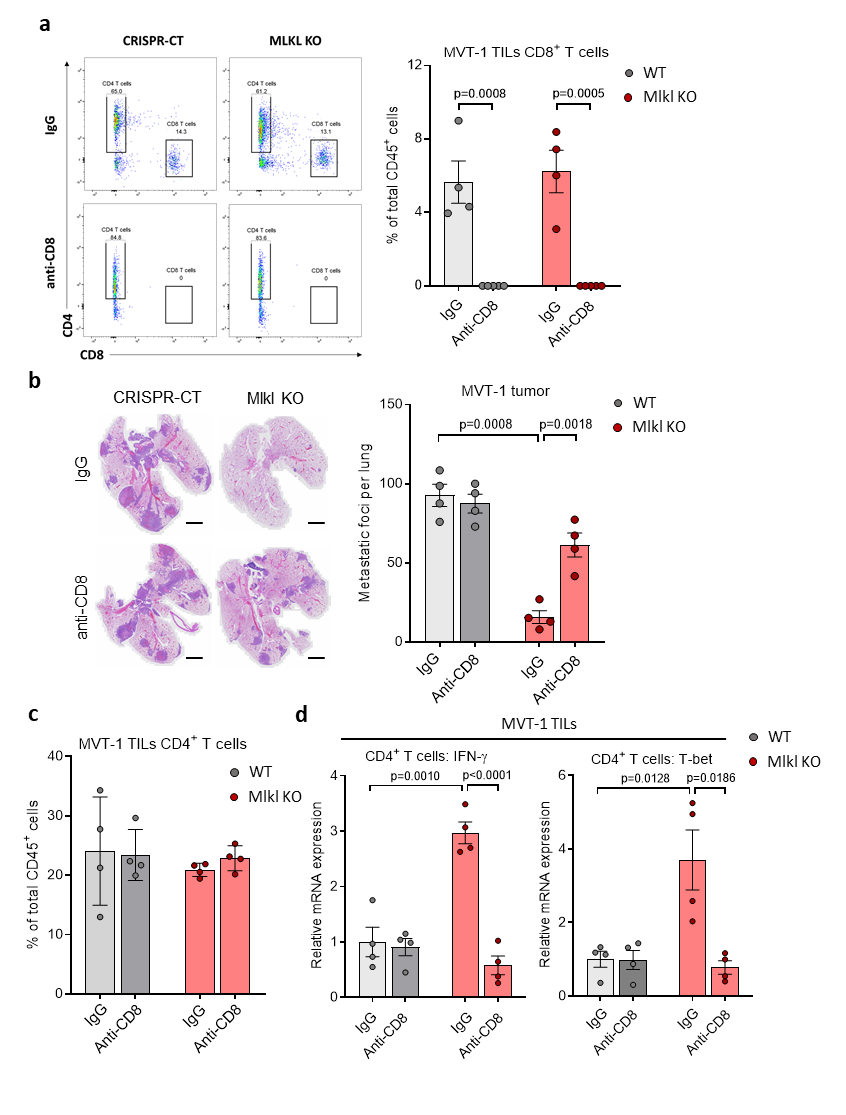
**

**Supplementary Figure S3. CD8+ T cells play important role in necroptosis-mediated tumor metastasis in MVT-1 tumor**. **a**, Right panel, Flow cytometry gating strategy for tumor-infiltrating CD8+ T cells. Left panel, flow cytometry analysis of tumor-infiltrating CD8+ T cells from mice at 5 weeks post-implantation with MVT-1-sgCT and MVT-1-Mlkl KO cells and treated with IgG or anti-CD8 antibody (n=4, each). **b**, Left panel shows the images of H&E stained lung sections from mice at 5 weeks post-implantation with MVT-1-sgCT and MVT-1-Mlkl KO cells and treated with IgG or anti-CD8 antibody. Scale bar, 2 mm. Right panel shows the quantification of metastatic foci in lungs (n=4, each). **c**, Flow cytometry analysis of tumor-infiltrating CD4+ T cells from mice at 5 weeks post-implantation with MVT-1-sgCT and MVT-1-Mlkl KO cells and treated with IgG or anti-CD8 antibody (n=4, each). No significant difference in p value was observed. **d**, qPCR analysis of T-bet and IFN-γ mRNA expression of tumor-infiltrating CD4+ T cells from mice at 5 weeks post-implantation with MVT-1-sgCT and MVT-1-Mlkl KO cells and treated with IgG or anti-CD8 antibody (n=4, each). Data presented as the mean ± sem and p value was determined by two-tailed t-test (**a**-**d**).

**Supplementary Figure S4.**

**
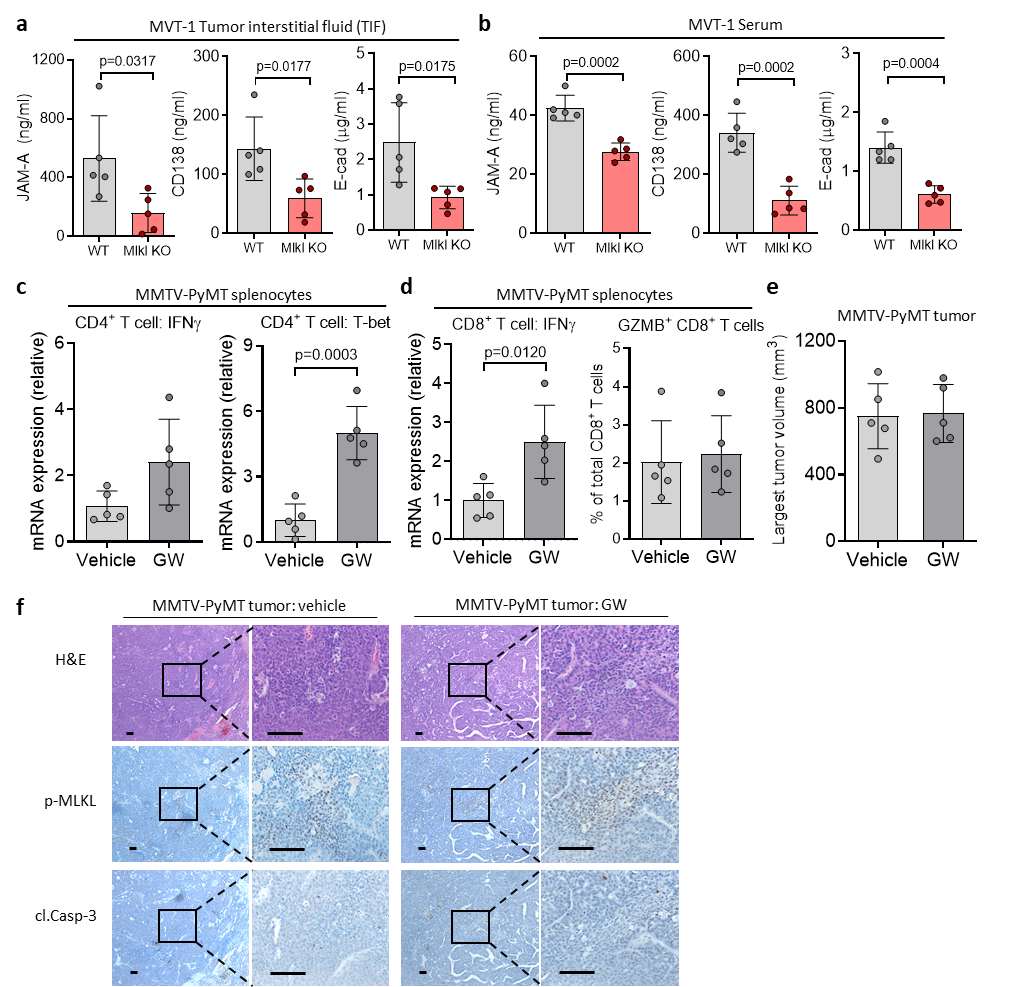
**

**Supplementary Figure S4. Necroptosis promotes tumor metastasis by activating cell surface proteases.**  **a**,**b** ELISA of soluble JAM-A, CD138 and E-cad in TIF (**a**) and serum (**b**) from mice at 5 weeks post-implantation with MVT-1-sgCT and MVT-1-Mlkl KO cells (n=5, each). **c**, qPCR analysis of T-bet and IFN-γ mRNA expression in spleen CD4+ T cells from MMTV-PyMT mice treated with Vehicle or GW280264X (n=5, each). **d**, qPCR analysis of IFN-γ mRNA expression in spleen CD8+ T cells from MMTV-PyMT mice treated with Vehicle or GW280264X (n=5, each; left panel). Flow cytometry analysis of spleen GZMB+ CD8+ T cells from MMTV-PyMT mice treated with Vehicle or GW280264X (n=5, each; right panel). **e**, Volume of largest tumor from MMTV-PyMT mice treated with Vehicle or GW280264X (n=5, each). No significant difference in p value was observed. **f**, Representative images of H&E and immunohistological stained tumor sections with phospho-MLKL (p-MLKL) or cleaved caspase-3 (cl.Casp-3) antibodies from MMTV-PyMT mice treated with Vehicle or GW280264X . Scale bar, 50 μm. Experiments were repeated three times, and representative data are shown. Data presented as the mean ± sem and p value was determined by two-tailed t-test (**a**-**e**).

**Supplementary Figure S5.**

**
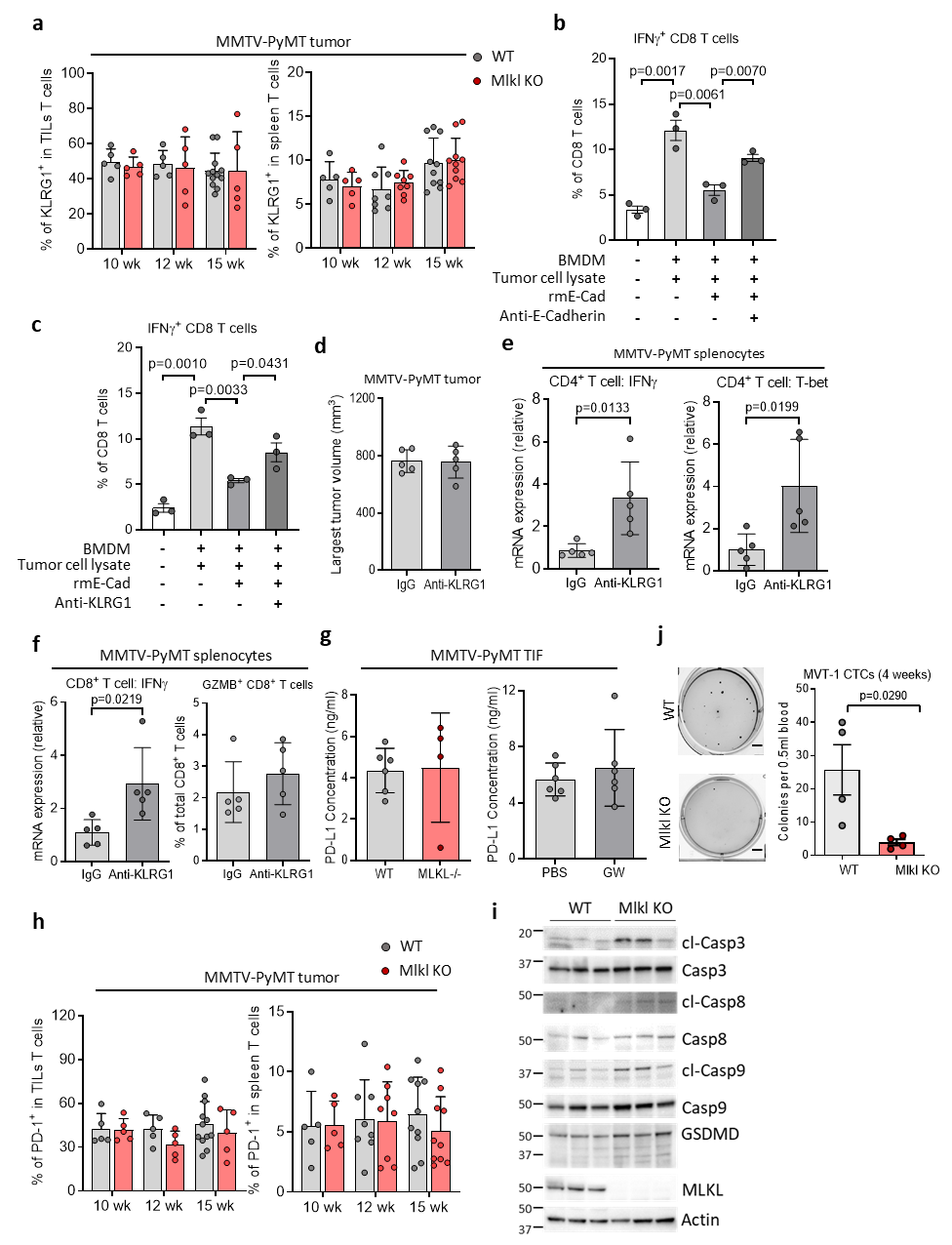
**

**Supplementary Figure S5. E-cad/KLRG1 pathway involved in tumor metastasis. a**, Flow cytometry analysis of tumor-infiltrating KLRG1+ T cells (left panel; 10 weeks, n=5, each; 12 weeks, n=5, each; 15 weeks, WT n=12, Mlkl KO n=5) and spleen KLRG1+ T cells (right panel; 10 weeks, n=5, each; 12 weeks, n=8, each; 15 weeks, n=10, each) from WT and Mlkl KO MMTV-PyMT mice. **b,c,** In vitro T cells activated by BMDM with LLC antigen following the treatments of rmE-cadherin and anti-E-cadherin antibody (**b**) or anti-KLRG1 antibody (**c**). **d**, Volume of largest tumor from MMTV-PyMT mice treated with IgG or anti-KLRG1 antibody (n=5, each). No significant difference in p value was observed. e, qPCR analysis of T-bet and IFN-γ mRNA expression in spleen CD4+ T cells from MMTV-PyMT mice treated with IgG or Anti-KLRG1 antibody (n=5, each). **f**, qPCR analysis of IFN-γ mRNA expression in spleen CD8+ T cells from MMTV-PyMT mice treated with IgG or Anti-KLRG1 antibody (n=5, each; left panel). Flow cytometry analysis of spleen GZMB+ CD8+ T cells from MMTV-PyMT mice treated with IgG or Anti-KLRG1 antibody (n=5, each; right panel). g, ELISA of soluble PD-L1 in TIF from WT and Mlkl KO MMTV-PyMT mice (Left panel, n=6, each) or MMTV-PyMT mice treated with Vehicle or GW280264X (right panel, n=6, each). No significant difference in p value was observed. **h**, Flow cytometry analysis of tumor-infiltrating PD-1+ T cells (left panel; 10 weeks, n=5, each; 12 weeks, n=5, each; 15 weeks, WT n=12, Mlkl KO n=5) and spleen PD-1+ T cells (right panel; 10 weeks, n=5, each; 12 weeks, n=8, each; 15 weeks, n=10, each) from WT and Mlkl KO MMTV-PyMT mice. No significant difference in p value was observed. **i,** Immunoblot analysis of tumor lysates from WT and Mlkl KO mice. Each group contain 3 mice samples. **j**, Left panel shows the representative images of crystal violet stained CTCs colonies from mice peripheral blood took at 4 weeks post-implantation with MVT-1-sgCT and MVT-1-Mlkl KO cells. Scale bar, 2 mm. Right panel shows the quantification of CTCs colonies (n=4, each). Data presented as the mean ± sem and p value was determined by two-tailed t-test (**a, d**-**h, j**). Data were pooled from three independent experiments, mean ± sem, two-tailed t-test (**b**, **c**).
